# Interactions of Methicillin Resistant *Staphylococcus aureus* USA300 and *Pseudomonas aeruginosa* in Polymicrobial Wound Infection

**DOI:** 10.1371/journal.pone.0056846

**Published:** 2013-02-22

**Authors:** Irena Pastar, Aron G. Nusbaum, Joel Gil, Shailee B. Patel, Juan Chen, Jose Valdes, Olivera Stojadinovic, Lisa R. Plano, Marjana Tomic-Canic, Stephen C. Davis

**Affiliations:** 1 Department of Dermatology and Cutaneous Surgery, Wound Healing and Regenerative Medicine Research Program, University of Miami Miller School of Medicine, Miami, Florida, United States of America; 2 Department of Pediatrics, University of Miami Miller School of Medicine, Miami, Florida, United States of America; 3 Department of Immunology and Microbiology, University of Miami Miller School of Medicine, Miami, Florida, United States of America; National Institutes of Health, United States of America

## Abstract

Understanding the pathology resulting from *Staphylococcus aureus* and *Pseudomonas aeruginosa* polymicrobial wound infections is of great importance due to their ubiquitous nature, increasing prevalence, growing resistance to antimicrobial agents, and ability to delay healing. Methicillin-resistant *S. aureus* USA300 is the leading cause of community-associated bacterial infections resulting in increased morbidity and mortality. We utilized a well-established porcine partial thickness wound healing model to study the synergistic effects of USA300 and *P. aeruginosa* on wound healing. Wound re-epithelialization was significantly delayed by mixed-species biofilms through suppression of keratinocyte growth factor 1. *Pseudomonas* showed an inhibitory effect on USA300 growth *in vitro* while both species co-existed in cutaneous wounds *in vivo*. Polymicrobial wound infection in the presence of *P. aeruginosa* resulted in induced expression of USA300 virulence factors Panton-Valentine leukocidin and α-hemolysin. These results provide evidence for the interaction of bacterial species within mixed-species biofilms *in vivo* and for the first time, the contribution of virulence factors to the severity of polymicrobial wound infections.

## Introduction

When the integument is compromised, bacterial microorganisms from the environment and skin surface are able to gain access to underlying tissues where the physical characteristics are optimal for colonization and growth. *Staphylococcus aureus* and *Pseudomonas aeruginosa* are among the most common organisms isolated from both acute and chronic wounds of various etiologies. Their prevalence has been demonstrated in surgical site infections as well as in the military setting where they have been attributed to causing infections of combat related injuries such as penetrating trauma and burn wounds [1], [2], [3], [4], [5]. Multiple studies have also emphasized the presence of bacteria and the polymicrobial nature of chronic, non-healing wounds [6], [7], [8], [9], [10], and the frequency of *S. aureus* and *P. aeruginosa* organisms has been shown to be exceedingly high [10], [11], [12], [13], [14]. This polymicrobial bioburden in wounds exists predominantly in the form of a biofilm resistant to antimicrobial treatments [15], [16], [17], [18], [19].

*S. aureus*, in its methicillin sensitive and methicillin resistant form (MRSA), is a common opportunistic pathogen, responsible for the majority of all superficial skin infections, resulting in increased morbidity, mortality, and tremendous health-care costs [20]. Increased incidence of MRSA-associated infections in both acute and chronic wounds is well-documented [3], [21], [22], and toxins as well as virulence factors produced by MRSA contribute in a major way to the pathogenicity [23]. These virulence factors include Panton-Valentine leukocidin (pvl), staphylococcal protein A (spa) and α-hemolysin (hla) [24]. The leading cause of community-associated bacterial infections in the United States is the MRSA isolate USA300 [25], which appears to have enhanced virulence as compared to the traditional hospital-associated MRSA strains [26], [27], [28]. While there has been an intense effort to better understand the mechanisms of MRSA virulence in various animal models, including skin abscesses [29], [30], the role of USA300 and its virulence during cutaneous wound healing has not been described.

The opportunistic pathogen *P. aeruginosa* expresses two types of quorum sensing (QS) population density-dependent systems, LasI-LasR and RhlI-RhlR. Both QS systems contribute to the pathology of cutaneous wound infections [31], [32], and LasI-LasR and RhlI-RhlR have been shown to regulate the expression of virulence factors such as exoenzyme S (ExoS) and exotoxin A (ToxA) which can further induce apoptosis in macrophages and neutrophils [33], [34], [35], [36].

The widespread presence of both USA300 and *P. aeruginosa* in acute and chronic wounds [3], [4], [5], [11], [12], the overwhelming incidence of staphylococcal skin and soft tissue infections, as well as the finding that wounds infected with *Pseudomonas* are slower to heal [31], [37], [38], all lend support to the notion that these two common pathogens are likely culprits in causing wound infection and delayed healing. Furthermore, infection with either *S. aureus* or *P. aeruginosa* has been shown to delay wound closure in mouse and rabbit wound healing models [31], [37], [39], [40], [41].

Current knowledge regarding the interactions between *S. aureus* and *P. aeruginosa* within polymicrobial wound infections is derived from both *in vitro* [42] and *in vivo* studies [39]. While *in vitro-*generated mixed species biofilms have been shown to delay healing when placed in a murine wound model [42], and a recent study demonstrated the ability of polymicrobial biofilms to impair healing in a rabbit ear model [39], to our knowledge, the role of bacterial virulence factors in polymicrobial wound infections has not been evaluated until now. In this study, we utilized a well-established porcine wound model [19], [43], [44] to investigate the effects of polymicrobial USA300 and *P. aeruginosa* infections. The *P. aeruginosa* strain used in the study was selected among different wound isolates based on the presence of QS systems, virulence factors and the ability to form a biofilm. Multi-species infected cutaneous wounds were compared to those containing only a single species or un-inoculated wounds, with respect to epithelialization, expression of bacterial virulence factors, and host response. Herein, we show that infection with both USA300 *and P. aeruginosa* significantly impairs wound closure as compared to single-species biofilms through down-regulation of keratinocyte growth factor 1 (KGF1) expression. In addition, we demonstrate induction of USA300 virulence factors *pvl* and *hla* in the presence of *P. aeruginosa*, providing *in vivo* evidence that interspecies interactions are operative in promoting bacterial pathogenicity and delayed healing in polymicrobial wound infections.

## Materials and Methods

### Bacterial Strains and Growth Conditions

Methicillin-resistant *S. aureus* USA300-0114 and 5 clinical combat wound isolates of *P. aeruginosa* (obtained from the U.S. Army Institute of Surgical Research, Fort Sam Houston, TX) were used. Tryptic soy broth (TSB) served as the growth medium, while Oxoid's Oxacillin Resistance Screening Agar Base (ORSAB) and *Pseudomonas* Agar with CN supplement (Oxoid) were used as selective media for plate-counting.

### *In vitro* Biofilm Assay

In order to quantify biofilm production by *P. aeruginosa* wound isolates, 10^6^ bacteria were inoculated in TSB media in 12-well polystyrene plates and incubated at 37°C for 24 h. Biofilm biomass by adherent bacteria was quantified upon removal of medium, washing with phosphate-buffered saline (PBS), and staining with 0.2% crystal violet [45]. Biofilm bound dye was recovered with 100 µl of 1% sodium dodecyl sulfate (SDS), and each biomass was quantified by measuring absorbance at 590 nm (*A*_590_). Wells incubated without bacteria were used as blanks. Alternatively, *P. aeruginosa* 09-010, USA300, or a combination of both species was grown in TSB under same conditions. In order to quantify the number of viable cells grown in the biofilm, bacteria were recovered with sonication at 50W for 10 sec to separate bacterial cells attached to the wells, and serial dilutions were plated on selective medium, ORSAB or *Pseudomonas* Agar with CN supplement, to determine colony forming units (CFU).

### Experimental Animals

Two young, female, specific pathogen-free pigs (SPF: Ken-O-Kaw Farms, Windsor, IL) weighing between 25 and 35 kg were used in this study. These animals were fed a non-antibiotic chow ad libitum before the study, fasted overnight before the procedures, and housed individually in our animal facilities (meeting USDA compliance) with controlled temperature (19–21°C) and controlled light and dark cycles (12 hour light/12 hour dark). The experimental animal protocols were approved by the University of Miami Institutional Animal Care and Use Committee and all the procedures followed the federal guidelines for the care and use of laboratory animals. In order to minimize possible discomfort, analgesics (buprenorphine and fentanyl transdermal patches) were used during the entire experiment. Forty eight (48) partial thickness wounds were made on the paravertebral area of each animal. The wounds were then divided into four groups, each containing 12 wounds. First group was inoculated with USA300, second group was inoculated with *P. aeruginosa,* third group was inoculated with both USA300 and *P. aeruginosa* simultaneously, and the fourth group remained un-infected as a control. Three wounds from each group were used to determine bacterial counts and bacterial RNA, and an additional three wounds were used for histological evaluation and host RNA isolation at days 2 and 4 post-wounding and inoculation from each animal.

### Wounding and Infection

Methods describing the animal preparation and wounding are explained in detail in our previous studies [43], [46]. Briefly, the flank and the back of experimental animals were prepped on the day of the experiment. Animals were anesthetized and partial thickness wounds (10 mm×7 mm) were made on the paravertebral area using a modified electrokeratome set at 0.5 mm deep [44]. The wounds were separated from one another by approximately 50 mm areas of unwounded skin.

Wounds were inoculated with 25 µl of 10^6^ CFU/mL of either USA300, *P. aeruginosa,* or both species immediately after wounding. In order to promote establishment of biofilm infection, wounds were covered with a polyurethane film dressing (Tegaderm; 3 M Health Care, St. Paul, MN) to allow for biofilm formation as previously described [43]. The film dressings were secured in place by wrapping the animals with self-adherent bandages (Petflex; Andover, Salisbury, MA).

### Bacterial Recovery and Quantification from Wounds

Three wounds from each treatment group at each assessment time were cultured quantitatively using a modified scrub technique [43]. Each wound was encompassed by a sterile surgical grade steel cylinder (22 mm outside diameter) and bacteria were collected by scrubbing the wound area with a sterile Teflon spatula into 1 ml of sterile phosphate buffer saline. 500 µl of collected suspension was centrifuged and the remaining pellet was preserved in RNAlater (Ambion) for RNA extraction. The remaining portion of the recovery suspension (500 µl) was used for the quantification of viable organisms. Serial dilutions were made and plated using a Spiral Plate System (Rockland, MA), and selective media, ORSAB for USA300 and *Pseudomonas* Agar with CN supplement was used to determine CFUs in the scrub solution collected from each wound. Plated bacteria were incubated aerobically for 24 hours at 37°C. CFUs were determined by the standard colony counting method.

### Biopsies and Histology

Three wounds per group were assessed on Days 2 or 4 post inoculation and wounding. A 4 mm punch biopsy was collected from the center of the wounds and stored in RNA later (Ambion) for further RNA isolation. Three additional punches were collected from unwounded skin and also preserved in RNA later. To evaluate wound healing rates, incisional biopsies from three wounds per condition from each animal were obtained; a transverse section approximately 3-mm thick was cut through the central part of the wound, including 5 mm of adjacent uninjured skin at day 2 and 4 post-wounding. The tissues were fixed and processed for paraffin embedding. 7 µm sections were cut and stained with hematoxylin and eosin. Staining was analyzed using a Nikon Eclipse E800 microscope, and the digital images were collected using SPOT camera advanced program. The wounds were quantified by planimetry as described previously [47], [48], [49].

### Immunohistochemistry

Paraffin sections were used for staining with anti-K14 antibody (1∶50, Abcam). 5 µm thick sections were de-waxed in xylene, rehydrated, and washed with 1× phosphate-buffered saline (PBS) (Fisher Scientific). For antigen retrieval, sections were heated in 95°C water bath in target retrieval solution (DAKO Corporation). The tissue sections were blocked with 5% bovine serum albumin (Sigma) and incubated with antibody against K14 overnight at 4°C. The slides were then rinsed in PBS, incubated with Alexa Fluor 488-conjugated goat anti-mouse antibody (Invitrogen) for 1 hr at room temperature and mounted with Prolong DAPI Gold antifade reagent (Invitrogen) to visualize cell nuclei. Specimens were analyzed using a Nikon eclipse E800 microscope and digital images were collected using the NIS Elements program.

### RNA Isolation

Porcine RNA was isolated using RNAspin Mini Kit (GE healthcare) following manufacturer’s instruction. Briefly, skin was digested with proteinase K treatment in TES buffer (30 mM Tris, 10 mM EDTA, 1% SDS, pH 8) and homogenized using a handheld homogenizer and disposable sterile pestle upon addition of RA1 lysis buffer. Bacterial RNA was isolated from both *in vitro* grown bacteria and collected wound scrub samples using RNeasy Mini Kit (Qiagen, Hilden, Germany). Bacterial pellets were digested and lysed in the presence of proteinase K containing either lysozyme (1 mg/ml; Sigma), or lysostaphin (Sigma, 30 U/ml) for samples collected from *P. aeruginosa* and USA300 wounds, respectively. Combination of lysozyme and lysostaphin was used for scrubs samples collected from mixed species inoculated wounds. RNA quality and concentration was assessed on the Agilent Bioanalyzer using the RNA 6000 Pico LabChip® Kit (Agilent Technologies, Waldbronn, Germany).

### cDNA Synthesis and Real-time qPCR

For real-time qPCR, 20 ng of total RNA from unwounded porcine skin and wound tissue was reverse transcribed and amplified using iScript One-Step RT-PCR Kit (Biorad). Real-time PCR was performed in triplicates using the IQ5 multi-color real-time PCR system (Bio-Rad). Relative expression was normalized for levels of GAPDH. The porcine primer sequences used were: GAPDH, forward (5′-ACATCATCCCTGCTTCTAC-3′) and reverse (5′-T TGCTTCACCACCTTCTTG-3′); IL-1α, forward (5′-GCCAATGACACAGAAGAAG-3′) and reverse (5′-TCCAGGTTATTTAGCACAGC -3′); IL-1β forward (5′-GGCTAACTACGGTGACAAC-3′) and reverse (5′- GATTCTTCATCGGCTTCTCC-3′); IL-6 forward (5′-TTCACCTCTCCGGACAAAAC-3′) and reverse (5′-TCTGCCAGTACCTCCTTGCT-3′); IL-8, forward (5′- GACCAGAGCCAGGAAGAGAC -3′) and reverse (5′-GGTGGAAAGGTGTGGAATGC-3′); IL-10 forward (5′-TGGAGGACTTTAAGGGTTAC-3′) and reverse (5′-CAGGGCAGAAATTGATGAC-3′); TNFα forward (5′-CACGCTCTTCTGCCTACTG-3′) and reverse (5′- ACGATGATCTGAGTCCTTGG -3′); KGF1 forward (5′-CAGTGACCTAGGAGCAACGA-3′) and reverse (5′-AAAGTGCCCACCAGACAGAT-3′).

For bacterial RNA, reverse transcription with DNase treatment was performed using QuantiTect Reverse Transcription Kit (Qiagen, Hilden, Germany) according to the manufacturers’ protocol and amplified using IQ Supermix (Bio-Rad). Gene quantification was performed in triplicates using the IQ5 multi-color real-time PCR system (Bio-Rad). For each sample, a mock reaction without addition of reverse transcriptase was performed. The sequences of the primers are shown in Table 1 and 2. Relative expression was normalized for levels of *gyrA* for USA300 and *rpoD* for *P. aeruginosa*. *GyrA*-FAM set of primers was used in combination with *hla*-CY5 and *spa*-HEX. Primers and probe for *pvl* gene were designed as previously described [50]. *P. aeruginosa* and USA300 specific primers and TaqMan probes were designed with the Beacon Designer™ software (Premier Biosoft International). The specificity of all primers was confirmed by sequencing of PCR products.

**Table 1:** *S. aureus* specific primer’s and probes sequences used for qPCR.

*Gene*	*Primer sequences*	*Probe*
*pvl* [50]	F: AATAACGTATGGCAGAAATATGGATGTR: CAAATGCGTTGTGTATTCTAGATCCT	ACTCATGCTACTAGAAGAACAACACACTATGG
*spa*	F: GTAACGGCTTCATTCAAAGTCTR: TCATAGAAAGCATTTTGTTGTTCT	AAAGACGACCCAAGCCAAAGCACT
*hla*	F: TCTGATTACTATCCAAGAAATTCG ATATTTCAGTGTATGACCAATCG	TTTGCACCAATAAGGCCGCC
*gyrA*	F: TGCTGAGTTAATGGAGGATATTGR:CACGTCTAATACCACTCTTACC	AGGTCCTGATTTCCCAACTGCT

**Table 2:** Sequences *of P. aeruginosa* specific primers used for PCR.

Gene	Forward primer	Reverse primer
*lasI*	ATCTGGGAACTCAGCCGTTTC	CGGACCGAAGCGCGATAC
*rhlI*	CCAGGGCATCTGCGGTTG	CTGCACAGGTAGGCGAAGAC
*algD*	GCCAACAAGGAATACATCGAGTC	GCCCAGCACCAGCACATC
*exoS*	CTGGATGCGGGACAAAAG	GTTCAGGGAGGTGGAGAG
*toxA*	CGACGTGGTGAGCCTGAC	GCTCCACCGTCCAGTTCTG
*rpoD*	AGAGAAGGACGACGAGGAAGAAG	GGCCAGGCCGGTGAGTTC

### Statistical Analysis

Statistical comparisons of CFUs were performed using Student’s t-test and presented as means±standard deviation (SDs). Statistically significant differences were defined as *p*<0.05. A comparison of epithelialization rates and gene-expression data for each treatment group was performed using GraphPad Prism 4. The means were analyzed by an ANOVA, followed by the Bonferroni post-hoc test. Significance was defined as a *p*<0.05.

## Results

### Differential Expression of Quorum Sensing Ligands and Virulence Factors in *P. aerugionsa* Wound Isolates

**Figure 1:**
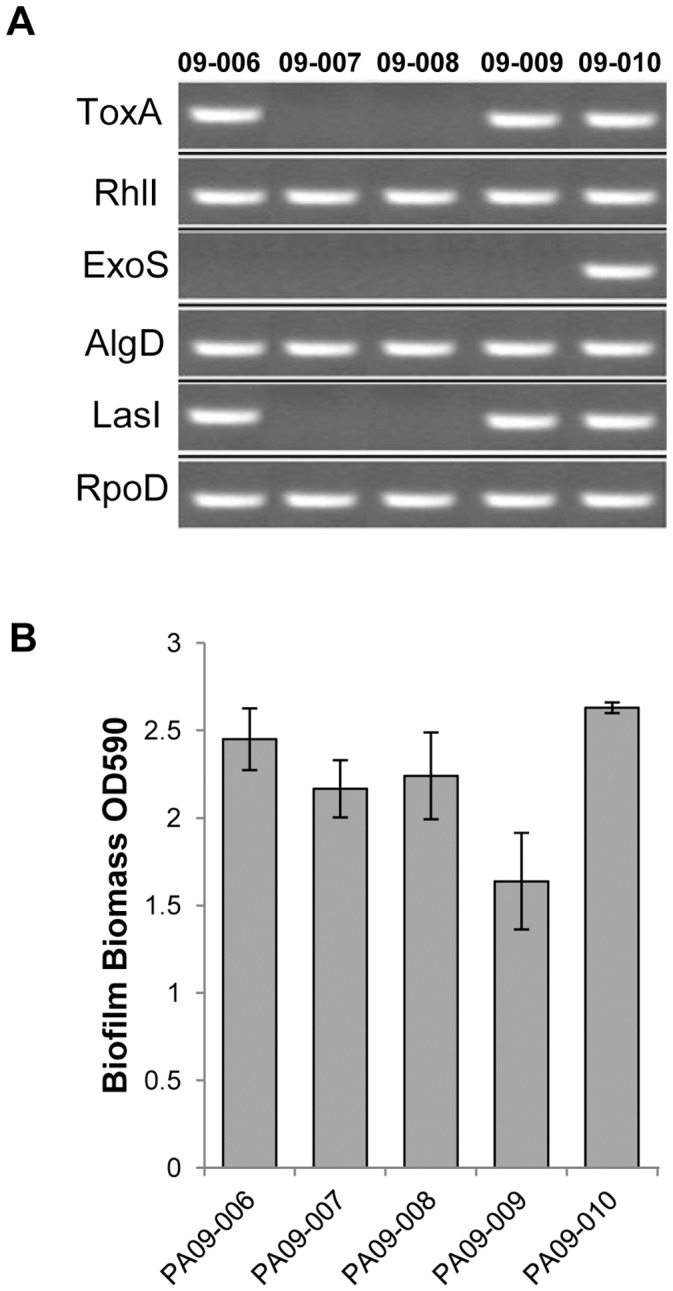
Differential expression of virulence factors, quorum sensing molecules and biofilm formation genes in five *P. aeruginosa* (PA) wound isolates. **A.** RT-PCR results showing expression of *ToxA*, *RhlI*, *ExoS*, *AlgD* and *LasI*; *RpoD* was used as a housekeeping gene. *P. aeruginosa* 09-010 isolate expressed all genes tested. **B.** *In vitro* biofilm assay by *P. aeruginosa* wound isolates. *P. aeruginosa* 09-010 has shown the most excessive biofilm production. The result is a summary of three independent experiments each repeated in triplicates.

To determine the presence and expression of virulence factors and quorum sensing molecules in six *P. aeruginosa* combat wound isolates we used PCR. Results revealed differences in expression of genes encoding quorum sensing ligands and *Pseudomonas* virulence factors in the wound isolates. Genes encoding autoinducer synthesis protein RhlI (*RhlI*) and a key enzyme in alginate production, GDP-mannose 6-dehydrogenase (AlgD), were present in all strains tested. Only one of the strains tested, *P. aeruginosa* 09-010, had a gene encoding *ExoS*, while the isolates 09-007 and 09-008 did not contain *ToxA* nor autoinducer synthesis protein LasI (**Fig. 1A**). Identical results were obtained when RNA was used as a template (data not shown). Of five isolates tested, only one *P. aeruginosa* 09-010, had all genes expressed. In addition *in vitro* biofilm assay identified isolate 09-010 as the most potent biofilm producer (**Fig. 1B**) and therefore this isolate was selected for further mixed-species studies with USA300.

### *P. aeruginosa* 09-010 Suppresses the Growth of USA300 *in vitro* and Less Efficiently *in vivo*

**Figure 2:**
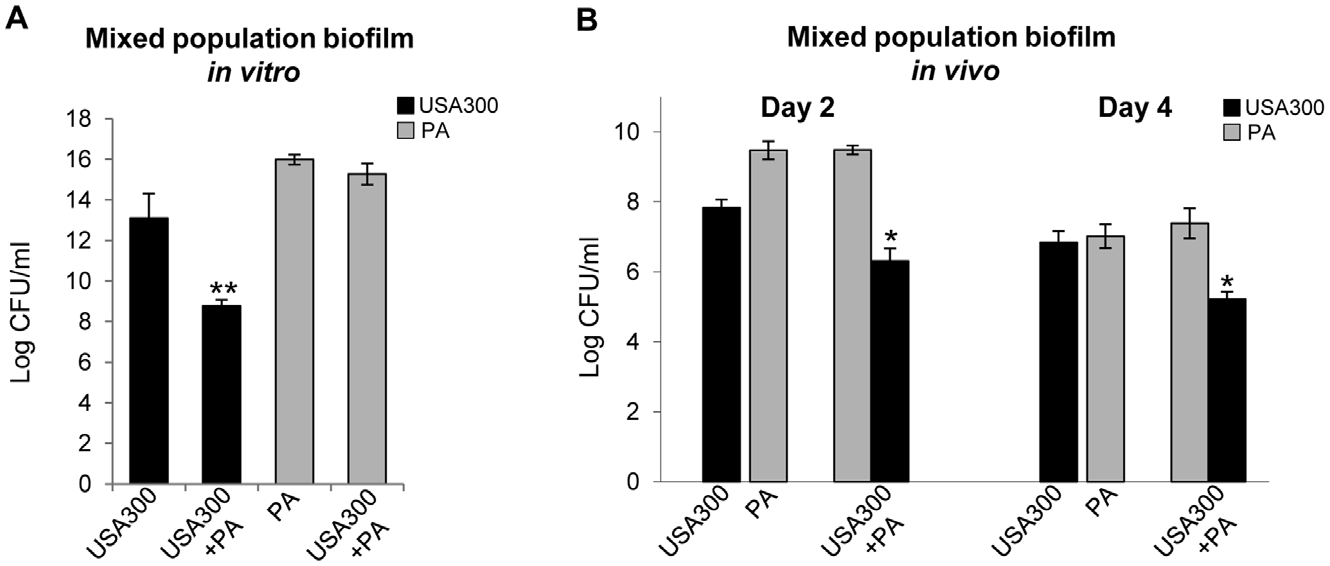
*P. aeruginosa* 09-010 supresses the growth of USA300 *in vitro* and *in vivo*. **A.** USA300 and *P. aeruginosa* 09-010 (PA) colony forming units (CFUs) were quantified upon growth of single-species or mixed species biofilms *in vitro* (n = 9). PA reduced the growth of USA300 by 4 logs (** *p*≤0.001). **B.** USA300 and *P. aeruginosa* 09-010 (PA) CFUs determined from porcine wounds on day 2 and 4 post infection (n = 6 per time point). *Pseudomonas* reduced the growth of USA300 (black bars) in the wounds infected by both species (* *p*≤0.05). The growth of *Pseudomonas* (grey bars) was not affected by the presence of USA300.

In order to assess the growth of USA300 and *P. aerugionsa* 09-010 in mixed-species biofilms, *in vitro* individual species and co-cultures were grown on polystyrene plates to allow for bacterial attachment and biofilm formation. USA300 and *P. aerugionsa* 09-010 CFUs were determined after removal of planktonic bacteria. In accordance to previous studies [51], [52] *Pseudomonas* had an inhibitory effect on *S. aureus* US300 growth, which was reduced by 4 logs in mixed-species biofilms *in vitro* (**Fig. 2A**).

To further determine the effects of mixed-species infection on the growth of individual species *in* *vivo*, bacteria were allowed 2 and 4 days post wounding to form a biofilm and CFUs were determined by plating scrubbed wound solution on selective media. Results show that USA300 and *P. aeruginosa* co-existed in the wounds; and that the growth of USA300 was slightly reduced by *P. aeruginosa* 09-010 *in vivo*, by 1.5 and 1.6 log on days 2 and 4 after wounding, respectively (**Fig. 2B**). The growth of *Pseudomonas* remained unchanged in the presence of USA300. Although with slightly reduced CFUs, both species remained present in wounds at day 4 post inoculation. There was no cross-contamination between the wounds as wounds inoculated with USA300 showed no detectable *Pseudomonas*, and no USA300 was recovered in *P. aeruginosa* 09-010 inoculated wounds. Neither *Pseudomonas* nor USA300 were detected in un-inoculated control wounds using selective growth media, and in addition, gram staining was used to document that control wounds remained non-infected. In summary, *Pseudomonas* has shown an inhibitory effect on USA300 growth in the mixed-species biofilms *in vitro,* while this effect was not as profound *in vivo.*

### Mixed-species Infection Delays Epithelialization through Suppression of KGF1

**Figure 3:**
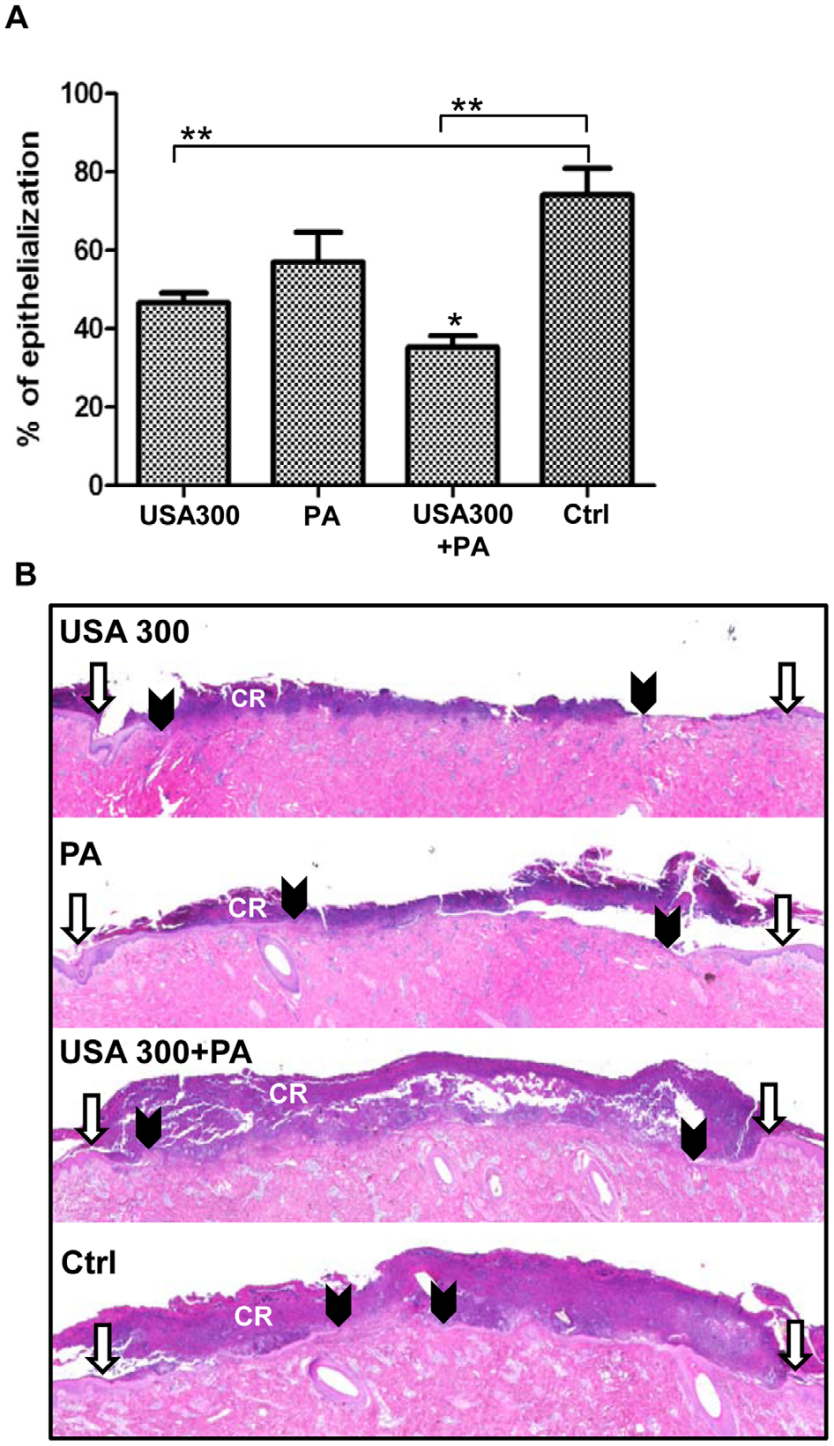
Mixed species biofilms inhibit epithelialization in porcine partial thickness wound model on day 2. A. Simultaneous infection with USA300 and *P. aeruginosa* 09-010 (PA) significantly inhibited epithelialization (* *p*≤0.05) when compared to wounds infected with single species or control (Ctrl) uninfected wounds (** *p*≤0.001). USA300 delayed epithelialization when compared to uninfected wounds (Ctrl) (** *p*≤0.001). **B.** Representative wounds stained with H&E are shown. White arrows indicate wound edges after initial wounding while black arrowheads point at the epithelialized edges of the migrating fronts. CR = crust.

Wound healing rates in single and mixed-species infected wounds were assessed by histology. The rate of epithelialization was measured using planimetry [47], [49]. Wounds with polymicrobial infection showed a significant wound healing impairment over the wounds infected with single-species biofilms (**Fig. 3A**)**.** Both USA300 and *P. aeruginosa* infected wounds showed delayed epithelialization when compared to control, uninfected wounds, however statistically significant differences were seen only in USA300 infected wounds (**Fig. 3B**). The average rate of epithelialization at day 2 for USA300, *P. aeruginosa,* and wounds infected with both species was 42%, 57%, and 35%, respectively, as compared to 74% wound closure in control uninfected wounds (**Fig. 3A**).

**Figure 4:**
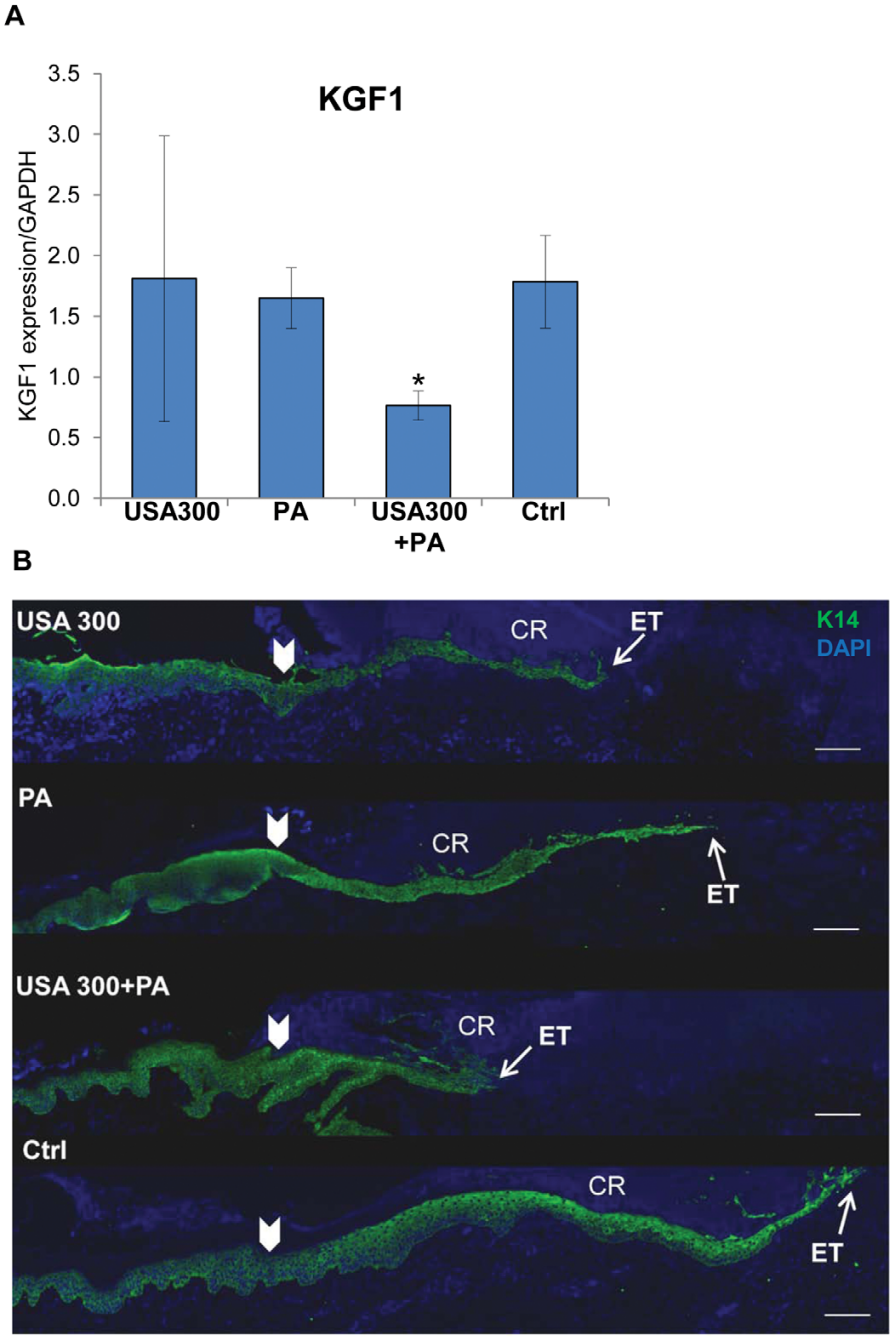
Mixed-species infection delays epithelialization through suppression of KGF1. **A.** Expression levels of KGF1 measured by qPCR in wounds infected with either USA300, *P. aeruginosa* (PA) or combination of both species (USA300+PA) and uninfected wounds (Ctrl). Mean values of expression levels were represented after normalization to GAPDH (n = 6). Error bars indicate mean SD. *statistically significant differences were defined as *p*<0.05. **B.** Immunolocalization of epidermal marker K14 (green) at the wound edge on day 2 post-wounding. Single-species and mixed-species infections inhibited epithelialization and decreased epithelial tongue (ET) thickness. CR = crust. White arrowheads indicate wound edges after initial wounding. Scale bar = 100 µm.

We further analyzed host response in an established porcine partial thickness wound healing model [19], [43], [46] using biopsies collected from the wound center after inoculation with *P. aeruginosa*, *USA300* or a combination of both bacterial species. Significant suppression of KGF1 was found in wounds infected by mixed-species biofilms (**Fig. 4A**), while single species infection did not result in suppression of KGF1 expression. KGF1 is produced during acute wound healing process mainly by fibroblasts, and has been shown to stimulate proliferation and migration of keratinocytes thus playing an important role in re-epithelialization [53], [54]. Staining with epidermal marker, K14 specific antibody, confirmed delayed epithelialization in infected wounds when compared to non-infected control wounds (**Fig. 4B**), with more pronounced inhibition of wound closure in mixed-species infected wounds. In addition K14 staining revealed reduced epithelial thickness in infected versus non-infected, control wounds (**Fig. 4B**).

### Immune Response in Single and Mixed-species Biofilm Wound Infections

**Figure 5:**
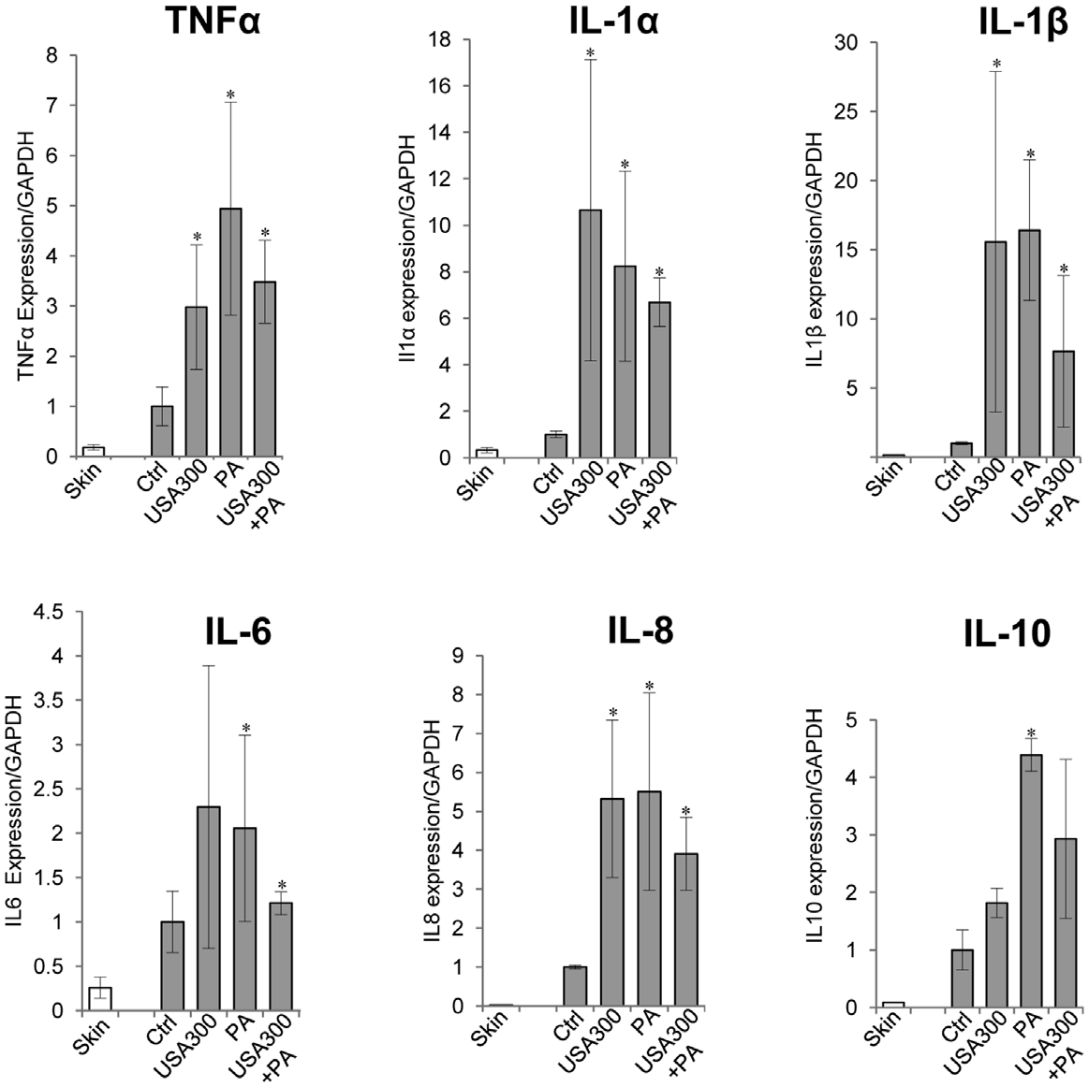
Single and mixed species induce distinct immune responses. Expression levels of TNFα, IL-1α, IL-1β, IL-6, IL-8, and IL-10 in wound tissue measured by qPCR in uninfected wounds (Ctrl), and wounds infected with either USA300, *P. aeruginosa* (PA) or combination of both species, USA300+PA. Expression levels in unwounded skin are also presented. Mean values of expression levels were represented after normalization to GAPDH (n = 6). Error bars indicate mean SD. *statistically significant differences were defined as *p*<0.05.

We also analyzed immune response at days 2 and 4 post-wounding to examine gene expression of pro- and anti-inflammatory cytokines [63] responding to single or mixed-species infection using qPCR. The most significant changes in cytokine expression were detected in wounds at day 2 post infection (**Fig. 5**). Significant induction was found in TNFα, IL-1α and IL-1β mRNA levels between infected and uninfected wounds, regardless of the bacterial species used for wound inoculation, suggesting that the presence of both USA300 and *P. aeruginosa* contributes to increased expression of these pro-inflammatory cytokines in cutaneous wounds. IL-8 expression was also induced in single and mixed-species infected wounds in comparison to uninfected wounds. Expression of IL-6 was induced in wounds infected with either mixed-species or *Pseudomonas.* IL-6 also showed an increasing trend in *USA300 infected wounds; however these changes were not significant in* comparison to control, non-inoculated wounds. The expression of IL-10, an anti-inflammatory cytokine, was significantly up-regulated in wounds infected with *Pseudomonas*, while USA300 or mixed-species infection did not cause significant induction of IL-10, although an increasing trend of IL-10 was observed in these wounds. We did not observe synergistic nor additive effects of polymicrobial infection on cytokine expression. In contrast, expression levels of IL-1α, IL-1β, IL-6 and IL-10 showed a decreasing trend in multi-species versus single-species infected wounds. These data show distinct differences in the host immune responses in porcine deep partial thickness wounds when stimulated with USA300, P. *aeruginosa,* or combination of both species. Although bacterial counts were still significantly high at day 4 post-wounding (**Fig. 2**) the expression of all tested cytokines was similar to expression levels detected in normal unwounded skin (data not shown). Even though infection inhibited early epithelialization, both uninfected and infected wounds epithelialized by day 4.

### Wound Environment and *P. aeruginosa* Presence Differentially Regulate Expression of USA300 Virulence Factors

**Figure 6:**
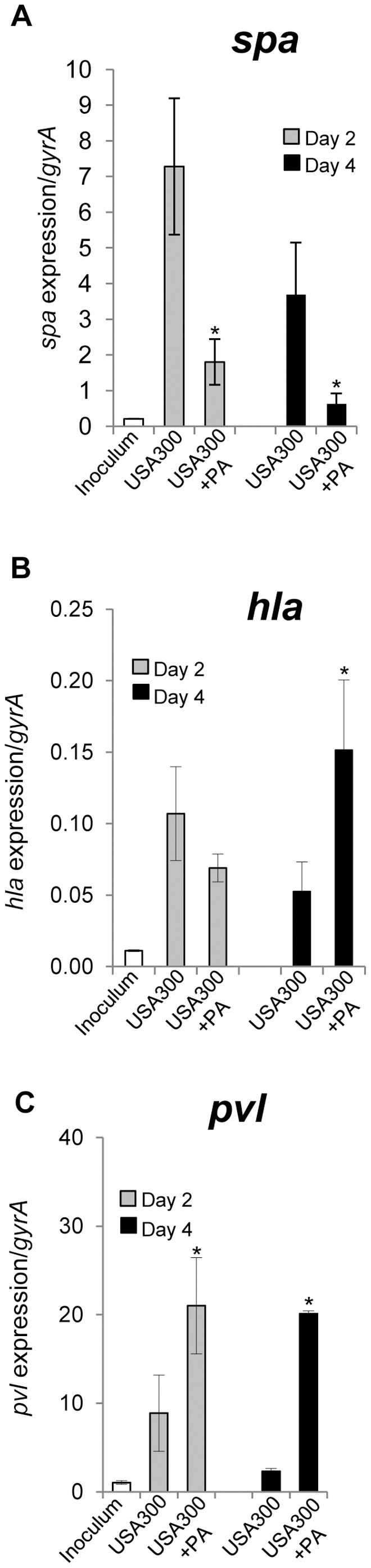
Expression of USA300 virulence factors in inoculated wounds. RT-PCR results for *spa* (A), *hla* (B) and *pvl* (C) expression in wounds colonized with single (USA300) and mixed species (USA300+PA) at days 2 and 4 post-infection (n = 6). The relative expression levels were normalized to *gyrA*. Inoculums = expression levels of *spa, hla* and *pvl* in USA300 culture used to infect the wounds. Error bars indicate mean SD. *statistically significant differences were defined as *p*<0.05.

To measure relative expression levels of USA300 virulence factors, *spa, hla* and *pvl*, RT-PCR analysis with bacterial RNA isolated from mixed- and single-species infected wounds was performed. Expression levels of virulence factors in the wound environment were also compared to mRNA levels of *spa, hla* and *pvl in vitro* (**Fig. 6**).The wound environment led to a striking induction of *spa* expression, with mRNA levels of *spa* 30 times higher in wounds as compared to *in vitro.* Interestingly, the presence of *P. aeruginosa* led to suppression of this virulence factor in the mixed-species colonized wounds at days 2 and 4 post-inoculation. In contrast to observed suppression of *spa*, the presence of *P. aeruginosa* induced expression of USA300 virulence factor *hla* on day 4 post wounding and infection (**Fig. 6**). The expression of *pvl*, a major cause of skin necrosis [55], [56], [57], was induced 8-fold in USA300 colonized wounds at day 2 versus *pvl* expression levels *in vitro*, while reduced *pvl* levels were seen on day 4 post wounding. More importantly the presence of *P. aeruginosa* in mixed species infected wounds resulted in a striking induction of *pvl* at both assessment times (**Fig. 6**).

## Discussion

The polymicrobial nature of non-healing wounds, such as venous, pressure, and diabetic foot ulcers is well documented and established [6], [7], [8], [9], [58], [59]. However, despite the exceedingly high frequency of *S. aureus* and *P. aeruginosa* in chronic and acute wounds, few *in vivo* studies have focused on the synergistic effects of these prevalent bacteria within the wound environment [39], [42]. In order to investigate functional consequences of polymicrobial infection with USA300 and *P. aeruginosa* on acute wound healing we utilized a well-established porcine skin wound model [19], [43], [46]. While murine [31], [37], [40], [41] and rabbit [39] models have previously been used to study the effects of bacterial infection on wound healing, the porcine model offers certain advantages such as its morphologic and physiologic similarity to human skin [60] as well as its large surface area which allows the creation of multiple wounds on a single animal that can be assessed at various time points.

One of the most important mechanisms by which USA300 and *P. aeruginosa* evade host immunity and establish persistent infections is via biofilm formation, and we have previously documented formation of bacterial biofilms in porcine cutaneous wounds [43]. In this study, we show that synergistic interactions between *P. aeruginosa* and USA300 delayed re-epithelialization by down-regulation of KGF1 compared to single species infected wounds (**Fig. 3** **and** **4**). Despite the fact that *P. aeruginosa* inhibited the growth of USA300 *in vitro* similarly to effects previously reported in murine and rabbit wound polymicrobial infection [42], in the porcine model, the wound environment allowed coexistence of both species and the growth of USA300 was only slightly reduced in comparison to the reduction observed *in vitro* (**Fig. 2**). This finding supports the fact that *Staphylococcus* is commonly isolated with *P. aeruginosa* from clinical samples, despite the multiple described mechanisms by which *Pseudomonas* virulence factors and exoproducts exert a negative influence on staphylococcal growth *in vitro* [52], [61], [62]. Our results suggest that the wound environment and increased virulence may protect USA300 from *P. aeruginosa* by a yet unidentified mechanism. The improved survival of USA300 in porcine wounds could possibly be due to the formation of staphylococcal small colony variants [52], [61], however further *in vivo* studies are needed to address this hypothesis.

Similarly to murine models of *S. aureus* wound infection [40], [41], inoculation of porcine wounds with USA300 alone was sufficient to delay early phases of wound closure as documented by K14 staining, while *Pseudomonas* alone demonstrated a less pronounced inhibition of epithelialization. Surprisingly, only polymicrobial infection resulted in a decreased expression of KGF1, suggesting the unique mechanism of wound healing inhibition by multi-species biofilms. Produced by fibroblasts, KGF1 acts in a paracrine fashion through the KGFR2IIIb receptor found exclusively on keratinocytes, resulting in increased migration and proliferation during wound healing [53]. Suppression of KGF1 together with decreased trend of IL-1α, IL-1β, IL-6 and IL-8 expression in mixed-species infected wounds when compared to wounds infected with a single species, indicates that polymicrobial infection delays wound closure through reduction of keratinocytes migration and proliferation rather than through increased inflammation. Our results contrast those of a recent study in a rabbit ear model where increased expression of pro-inflammatory cytokines TNFα and IL-1β was seen with polymicrobial biofilm infection [39] and these differences may be explained by the variability between bacterial strains and animal models utilized.

Our study has also shown the lack of *Pseudomonas* virulence factors and genes for QS molecules within wound isolates, which goes in line with previous studies [64] confirming that spontaneous QS- and virulence-deficient *P. aeruginosa* mutants are still capable of causing wound infections. *Pseudomonas* has two QS systems, LasI-LasR and RhlI-RhlR, shown to be important for wound infection and biofilm formation in rodent models [32], [36]. Although we analyzed a small number of isolates, the lack of *LasI* in some and the presence of *Rh*lI in all of the tested strains suggest that RhlI might be more important than LasI for cutaneous wound infections. Furthermore, the presence of AlgD, a key enzyme in alginate production, in all strains indicates its importance in establishing wound infection, while further studies are needed in order to fully characterize the roles of *exoS* and *ToxA* during cutaneous wound healing.

Lastly, our data revealed that synergy within mixed-species wound biofilms significantly increased the virulence of USA300. The presence of *P. aeruginosa* within the polymicrobial wound environment resulted in a marked increase of *pvl and hla* expression suggesting an overall induction of USA300 virulence and its detrimental effects on wound healing in polymicrobial infections. The importance of *pvl* in *S. aureus* infections is mostly controversial mainly due to the limitations of the widely used murine models [65], [66], [67], [68], [69], as neutrophils, the primary target cells for *pvl*, are highly insensitive to this pore-forming toxin in mice [68], [70], [71]. In addition, *S. aureus* cannot fully utilize iron from murine hemoglobin [68], [70], [71]. Recent studies showing the importance of *pvl* in skin necrosis [55] confirmed that alternative animal models are more appropriate for studying *S. aureus* skin infection and therefore the porcine wound model due to its many similarities to wound healing in humans offers multiple advantages. Our results demonstrating induction of *hla in* *vivo* versus *in vitro,* regardless of *P. aeruginosa* presence support a recent *in vitro* study that showed the importance of *hla* for invasion through human keratinocytes [72]. The same study indicated that *pvl* and *spa* were not important for USA300 penetration through intact epidermis [72], however this data cannot be fully extrapolated to the wound healing process where the epidermal barrier is compromised, suggesting that the role of *pvl* and *spa* in wound healing remains to be fully elucidated. In contrast to the observed induction of *pvl and hla,* our study showed that polymicrobial infection led to suppression of *spa*. Nevertheless, this virulence factor was strikingly induced in USA300 infected wounds when compared to *in vitro* expression levels, suggesting its possible role during the initial phases of wound infection. Taken together, future studies using mutant *S. aureus* strains lacking individual virulence factors in the porcine model are needed to address the questions regarding the role of USA300 virulence factors in inhibition of wound healing.

Our data underline the importance of bacterial interactions in multi-species wound infections as we demonstrate that bacterial synergy can alter virulence, delay healing and perhaps even change susceptibility of microbial communities to antimicrobial therapy. Given the fact that chronic wounds are infected with more than one species, understanding of interspecies interactions is imperative for developing new successful therapeutic approaches against polymicrobial wound infections.
